# New control of the senescence barrier in breast cancer

**DOI:** 10.1080/23723556.2019.1684129

**Published:** 2020-01-20

**Authors:** Tânia D. F. Costa, Staffan Strömblad

**Affiliations:** Department of Biosciences and Nutrition, Karolinska Institutet, Huddinge, Sweden

**Keywords:** Cellular senescence, p21-activated kinase, PAK4, RELB, CEBPB

## Abstract

Normal cells exposed to cancer-causing events respond by triggering cellular senescence, a stress response which halts cell proliferation and constitutes a protective anti-cancer barrier. We have uncovered a previously unknown signaling pathway implicating p21-activated kinase 4 (PAK4) in the control of senescence in breast cancer, via the nuclear factor kappa-light-chain-enhancer of activated B cells (NF-κB) subunit RELB and the CCAAT-enhancer-binding protein beta (C/EBPβ).

Normal cells subjected to oncogene activation undergo cellular senescence, which imposes a distinctive growth arrest that potently prevents cancer, a subject in focus of our recent study.^1,^^2^ The capacity to overcome this senescence barrier has been postulated as a crucial step in the progression from pre-malignant to malignant tumors. Consistently, senescent cells are abundant in pre-malignant lesions of various cancers but rare in subsequently established tumors.^2,3^ However, a senescence response may be restored in established cancer cells.^3^ Senescent cells exhibit a secretome that can both reinforce the growth arrest via autocrine signaling and influence the behavior of neighboring cells via paracrine signaling in beneficial ways (*i.e*. inducing senescence or attracting immune cells for self-clearing) or detrimental ways (*i.e*. contributing to malignant transformation of nearby cells or triggering cancer promoting inflammation).^4^ Importantly, recent studies have highlighted the possibility of combinatorial treatments that successfully first induce a senescence response and then selectively induce their killing.^4^

The serine/threonine p21-activated kinases (PAKs) are key cell signaling hubs with proven roles in several of the hallmarks of cancer.^5^ Specifically, p21-activated kinase 4 (PAK4) can confer apoptosis resistance and anchorage-independent cell growth and regulate important aspects of cell proliferation, cell migration and drug-resistance.^6^ PAKs are frequently overexpressed in cancer, often correlated to poor patient outcome.^6^

While a role for PAK4 overexpression in breast cancer has been suggested^6^ this hypothesis has remained untested *in vivo*. To this end, we developed a mouse model with PAK4 overexpression in the mammary epithelium under the control of the MMTV-LTR (mouse mammary tumor virus long terminal repeat) promoter, mimicking the PAK4 overexpression found in breast cancer. Nulliparous PAK4-overexpressing mice developed mammary lesions by 6 months of age that progressed into mammary tumors in 25% of the cases at 20–24 months of age.^1^ Importantly, exome sequencing revealed that these tumors occasionally exhibited activating RAS mutations. Normal cells respond to oncogenic RAS by activating the cellular senescence program that blocks further growth of these damaged cells and thus acts as a vital barrier to tumorigenesis.^2^ Interestingly, expression of constitutively active PAK4 in mouse embryonic fibroblasts induced a similar premature senescence phenotype, suggesting that hyper-activated PAK4 may act as an oncogene.^7^ However, the relatively low penetrance and late tumor appearance in mammary glands upon wild-type PAK4 overexpression in combination with these tumor’s inherent RAS mutations suggests that wild-type PAK4 may not constitute a strong mammary oncogene *per se*, but rather acts as a facilitator of tumorigenesis driven by other oncogenes, including mutant RAS.^1^

We therefore hypothesized that PAK4 overexpression may overcome the RAS-induced senescence barrier. Indeed, upon PAK4 overexpression, human mammary epithelial cells (HMECs) with inducible H-RAS-V12 expression no longer displayed the typical senescence-associated growth arrest, but instead retained some proliferative capacity.^1^ This shows that PAK4 confers a selective advantage to oncogenic RAS-expressing cells to overcome the senescence barrier and thereby may selectively contribute to subsequent tumors (Figure 1(a)). This finding might have important repercussions given the widespread prevalence of oncogenic RAS mutations in cancer (found in nearly 30% of all human cancers and in over 90% of pancreatic cancers) and the challenges in pharmacologically targeting mutant RAS.^9^

**Figure 1.**
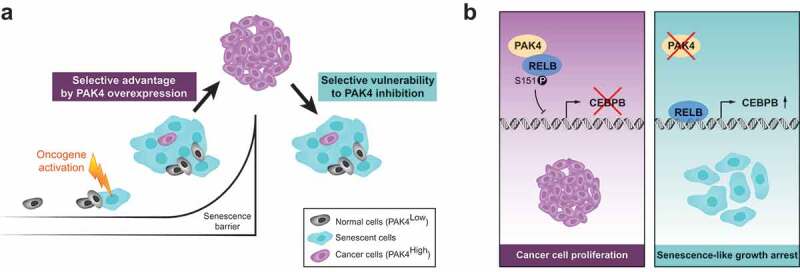
New signaling pathway controlling the senescence barrier in breast cancer. **(a)** In untransformed human mammary epithelial cells with low PAK4 expression levels (gray cells), exposure to oncogenes causes oncogene-induced senescence (OIS, blue cells), a major barrier to cancer development. However, we found that PAK4 overexpression can override the OIS barrier in mammary epithelial cells, indicating a route by which PAK4 may promote cancer, consistent with the commonly observed PAK4 overexpression in cancer (purple cells). We also found that PAK4 inhibition in established breast cancer elicits a senescent-like growth arrest, indicating a vulnerability of breast cancer that may be utilized for the development of therapy. **(b)** A PAK4 – RELB - C/EBPβ axis mechanistically controls the senescence phenotype in breast cancer. Abundant PAK4 interacts with and phosphorylates serine 151 of RELB (S151), impairing RELB DNA-binding and transcription, including of C/EBPβ, thereby facilitating cancer cell proliferation. When PAK4 is inhibited, RELB accumulates, binds to DNA and promotes C/EBPβ expression, leading to a senescence-like growth arrest. Both panels are based on our recent results^1^. The schematic in Panel A is from Costa 2019^8^ with permission, inspired by Narita & Lowe.^3^ Panel B as in original from Costa *et al*., 2019^1^. PAK4: p21-activated kinase 4; C/EBPβ: CCAAT-enhancer-binding protein beta; RELB: nuclear factor kappa-light-chain-enhancer of activated B cells (NF-κB) subunit.

With this in mind, we tested if PAK4-inhibition could restore senescence in established cancer. Indeed, we observed a senescence-like response upon PAK4 inhibition *in vitro, in vivo* and *ex vivo* using a variety of models (cellular and animal models) and tools (siRNA, CRISPR/Cas9 and small compound inhibition) (Figure 1(a)).^1^ Upon PAK4 depletion, a panel of breast cancer cells lines showed senescence associated (SA) features including morphological changes, increased SA-β-galactosidase activity combined with inhibited cell proliferation and gene expression changes consistent with a SA-phenotype. This was also observed in an extended panel of cancer cells lines originating from pancreatic and ovarian cancer and in primary cells derived from the transgenic PyMT (Polyoma Middle T) breast cancer mouse model and a KRAS-driven model of pancreatic cancer, as well as in primary cancer cells derived from breast cancer patients. Importantly, normal primary HMECs, expressing low PAK4 levels, did not undergo senescence upon PAK4 knockdown, indicating the possibility of selective targeting of cancer cells. *In vivo*, PyMT-driven mammary tumorigenesis was compared between mice with or without MMTV-Cre recombinase mediated conditional PAK4 gene depletion. Analyzes of early stage mammary glands harvested from PAK4-depleted females exhibited significantly less hyperplasia and mammary lesions compared to control mice. Accordingly, palpable mammary tumors appeared considerably later in PAK4 knock-out mice that also presented an extended overall survival. Importantly, an increase in SA-β-galactosidase activity was detected in lesions of PAK4-depleted mammary glands as compared to controls. Additionally, pharmacological treatment of established PyMT tumors with PF-3758309 increased SA-β-galactosidase positive tissue areas. Further, human breast cancer cells harboring stable PAK4 knockdown grew slower when injected subcutaneously onto the back of immunodeficient mice and formed smaller tumors that were highly positive for SA-β-galactosidase activity.^1^ We could thus show that several epithelial cancers are susceptible to arrest upon PAK4 inhibition, as suggested for glioblastomas^10^ further supporting the notion of generalized PAK4 addiction in cancer.^5^

Guided by transcriptome analyzes, mass spectrometry and computational modeling, we also identified a new PAK4-mediated signaling pathway that controls senescence in breast cancer (Figure 1(b)).^1^ The nuclear factor kappa-light-chain-enhancer of activated B cells (NF-κB) subunit RELB was functionally required for the growth arrest observed upon PAK4 inhibition. While the role of canonical NF-κB in senescence is well established (as many of the senescence associated secretory phenotype genes are NF-κB targets), the role for non-canonical NF-κB subunits has remained elusive.^11^ Importantly, we found that PAK4 interacts with and phosphorylates RELB at serine 151, thereby inhibiting RELB DNA-binding and consequential transcription, including of the CCAAT-enhancer-binding protein beta (C/EBPβ).^1^ Consistently, inhibition of PAK4 sparked the senescent phenotype at least in part through the upregulation of C/EBPβ.^1^ Importantly, we also extended our findings to large clinical datasets of breast cancer patients where we found PAK4 overexpression across all breast cancer subtypes and *PAK4* amplification in a small subset of patients, both correlated to poor patient outcome.^1^

Based on this and on our findings that PAK4 inhibition restored senescence in breast cancer, PAK4 inhibition may be explored as a potential therapeutic strategy for breast cancer, the most frequently diagnosed cancer in women world-wide and one of the major causes of lethality.^12^
